# Differentiated evolutionary rates in alternative exons and the implications for splicing regulation

**DOI:** 10.1186/1471-2148-6-50

**Published:** 2006-06-22

**Authors:** Mireya Plass, Eduardo Eyras

**Affiliations:** 1 Research Unit of Biomedical Informatics, IMIM – Pompeu Fabra University, E08003, Barcelona, Spain; 2 Catalan Institution for Research and Advanced Studies (ICREA), E08010, Barcelona, Spain

## Abstract

**Background:**

Alternatively spliced exons play an important role in the diversification of gene function in most metazoans and are highly regulated by conserved motifs in exons and introns. Two contradicting properties have been associated to evolutionary conserved alternative exons: higher sequence conservation and higher rate of non-synonymous substitutions, relative to constitutive exons. In order to clarify this issue, we have performed an analysis of the evolution of alternative and constitutive exons, using a large set of protein coding exons conserved between human and mouse and taking into account the conservation of the transcript exonic structure. Further, we have also defined a measure of the variation of the arrangement of exonic splicing enhancers (ESE-conservation score) to study the evolution of splicing regulatory sequences. We have used this measure to correlate the changes in the arrangement of ESEs with the divergence of exon and intron sequences.

**Results:**

We find evidence for a relation between the lack of conservation of the exonic structure and the weakening of the sequence evolutionary constraints in alternative and constitutive exons. Exons in transcripts with non-conserved exonic structures have higher synonymous (dS) and non-synonymous (dN) substitution rates than exons in conserved structures. Moreover, alternative exons in transcripts with non-conserved exonic structure are the least constrained in sequence evolution, and at high EST-inclusion levels they are found to be very similar to constitutive exons, whereas alternative exons in transcripts with conserved exonic structure have a dS significantly lower than average at all EST-inclusion levels. We also find higher conservation in the arrangement of ESEs in constitutive exons compared to alternative ones. Additionally, the sequence conservation at flanking introns remains constant for constitutive exons at all ESE-conservation values, but increases for alternative exons at high ESE-conservation values.

**Conclusion:**

We conclude that most of the differences in dN observed between alternative and constitutive exons can be explained by the conservation of the transcript exonic structure. Low dS values are more characteristic of alternative exons with conserved exonic structure, but not of those with non-conserved exonic structure. Additionally, constitutive exons are characterized by a higher conservation in the arrangement of ESEs, and alternative exons with an ESE-conservation similar to that of constitutive exons are characterized by a conservation of the flanking intron sequences higher than average, indicating the presence of more intronic regulatory signals.

## Background

Alternative splicing (AS) can have a biologically relevant effect on protein structure, as it allows the shuffling of protein domains rather than disrupting them [1]. Consequently, alternative splicing can modulate the function of a gene, affecting, for instance, the signal peptides and the transmembrane segments [2,3]. The importance of AS in many genomes has raised the question of its role in the context of evolution. Modrek and Lee [4] have proposed AS as an evolutionary mechanism that gives an organism the possibility to explore new protein functions by allowing the addition of novel domains while maintaining the rest of a protein intact. This has suggested that alternative exons may have more freedom to change its amino acid sequence. Indeed, recent reports show that conserved alternative exons have higher dN than average [5-8], and can even have higher dS than average [8]. However, the opposite effect has also been observed: alternative exons have been reported to have a DNA sequence conservation higher than average [9,10]. The higher conservation has been attributed to the fact that alternative exons are in general more regulated than constitutive ones, and therefore contain more conserved sequence motifs, like exonic splicing enhancers and silencers, which function in a coordinated fashion. The conservation of these motifs is important for exon definition [11], and in some cases a single nucleotide mutation can disrupt the splicing and lead to a disease state, like dementia [12] or spinal muscular atrophy [13]. In fact, exonic regions with high density of regulatory motifs have been linked to regions of low SNP density [14], low synonymous SNP density [15], and negative selection against synonymous substitutions [16-18].

Orthologous exons with similar splicing regulation show sequence conservation of the cis-acting motifs [18]. On the other hand, it is known that cis-acting regulators of splicing are sometimes not conserved in sequence between orthologous genes [19] or are not located at orthologous positions [20], but still can preserve their function. Furthermore, regulatory elements can function at different distances from the splice sites. This distance has been found to influence the strength of the splicing regulator [21], and there seems to be a distance beyond which a motif becomes inactive [22] or changes its regulating activity [23]. We therefore expect that if two orthologous exons have the same regulation, the cis-acting motifs responsible for this regulation should also show some positional conservation. Conversely, two orthologous exons with different regulation should show low conservation in sequence and/or position of the regulatory motifs involved in splicing. Additionally, as mentioned before, a variation in the sequence and/or the arrangement of the regulatory elements is found to affect the splicing pattern, hence we expect that an arrangement of regulatory elements that is not conserved between orthologous exons must be related to a lack of conservation of the exonic structure of the transcripts including that exon. The mRNA produced from the pre-mRNA is determined by the exonic and intronic signals regulating its splicing, hence the conservation of these signals during evolution implies a conservation of the definition of the exon-intron boundaries. Conversely, transcripts that do not conserve the exonic structure across evolution must be defined by splicing regulatory signals that are not conserved in sequence and/or arrangement. Based on these observations we expect orthologous exons, alternative or constitutive, participating in transcripts with non-conserved exonic structure to have less sequence conservation. A recent report shows that constitutive exons in transcripts with non-conserved exonic structure have greater non-synonymous substitution rate [24]. In this work we generalize these results. We provide further evidence for the differences in sequence evolution between alternative and constitutive exons and link these differences to the pattern of conservation of the exonic structure. We also define a measure of the conservation of the arrangement of exonic splicing regulators and study the variation of this arrangement and the relation to the sequence divergence of exons and introns.

## Results and discussion

### Exonic structure and the sequence evolution of exons

We considered a set 1211 human and mouse orthologous genes containing conserved constitutively and alternatively spliced exons. From these we selected 2133 exons that were alternative in human and mouse and 8788 exons that were constitutive in human and mouse (see Methods for details). For this set we found lower synonymous substitution rates (dS) (Kolmogorov-Smirnov (KS) test p-value < 2.2e-16) and higher non-synonymous substitution rates (dN) (KS test p-value < 2.2e-16) for alternative exons compared to constitutive ones. We also found that alternative exons have higher values for omega (= dN/dS) than constitutive ones (KS test p-value < 2.2e-16). We tested these results by performing the comparisons in a different approach. We concatenated, for every gene, all constitutive exons into what we called a constitutive region, and all alternative exons into an alternative region, and compared the dN, dS and omega values in both regions within each gene. We found significant differences in dS (Wilcoxon signed-ranks test p-value < 2.2e-16) and dN (Wilcoxon signed-ranks p-value = 1.140e-5) between alternative and constitutive regions. Figures S1 and S2, provided as supplementary material [see Additional File 1], show lower dS values and higher dN values for alternative regions. Additionally, we found a significant difference for omega (Wilcoxon signed-ranks test p-value < 2.2e-16) and Figure S3 [see Additional File 1] shows higher omega values for alternative regions compared to constitutive regions. These results are in agreement with previous reports [5-7].

We separated the constitutive and alternative exons into four groups according to whether they were part of a transcript with an exonic structure that is conserved in mouse or not (see Methods for the details of this classification). Exons in transcripts with conserved exonic structure (CES) are called CES exons, whereas exons in transcripts with non-conserved exonic structure are called non-CES exons. A non-CES exon is such that there is a pattern of splicing of the pre-mRNA, which includes this exon, and which is never the same in the orthologous mRNA when the orthologous exon is included.

**Figure 1:**
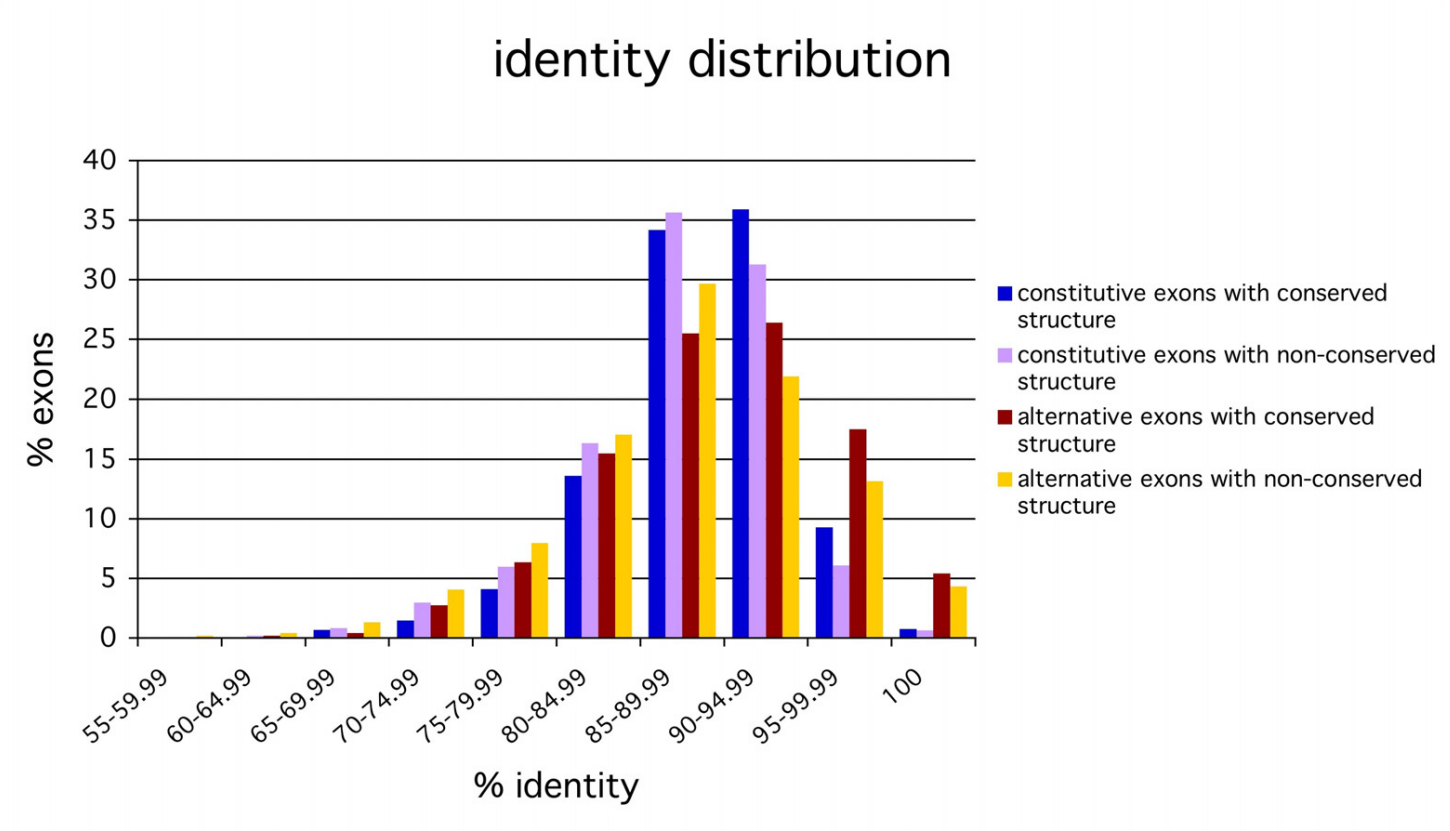
Distribution of the percentage identity conservation for each of the four subsets of orthologous exons. The four exon subgroups are defined according to whether the exon is constitutive or alternative, and whether it is part of a transcript with a conserved exonic structure (CES) or not (non-CES).

Analysis of the sequence identity conservation of these four groups revealed statistically significant differences between the four distributions (see Table 1 for p-values). These distributions show that alternative exons are overrepresented in the high and low conservation ranges with respect to constitutive exons (Figure 1). Alternative CES exons are overrepresented in the range of high sequence conservation, whereas alternative non-CES are more abundant in the range of low conservation (Figure 1). On the other hand, constitutive non-CES exons are more frequent in the low conservation range than constitutive CES exons, which are more frequent in the high conservation range. Even though the distributions are different, the average identity conservation is very similar between groups: 88.83% (median 89.47%) for constitutive CES exons, 87.67% (median 88.51%) for constitutive non-CES exons, 89.28% (median 89.81%) for alternative CES exons, and 87.76% (median 88.39%) for alternative non-CES exons. Thus alternative CES exons have the highest average conservation, whereas alternative non-CES exons have an average conservation similar to constitutive exons.

**Table 1:** P-values for the exon-based comparisons of percent identity, dN, dS and omega.

	Identity	dN	dS	omega
C CES vs C non-CES	< 2.2e-16	< 2.2e-16	0.004262	< 2.2e-16
C CES vs A CES	3.505e-13	1.057e-13	< 2.2e-16	< 2.2e-16
C CES vs A non-CES	4.569e-13	< 2.2e-16	< 2.2e-16	< 2.2e-16
A CES vs C non-CES	< 2.2e-16	0.0003056	< 2.2e-16	3.345e-12
A CES vs A non-CES	6.62e-06	5.795e-05	0.02868	0.002465
C non-CES vs A non-CES	4.013e-10	1.165e-13	< 2.2e-16	< 2.2e-16

This table contains the p-values for the Kolmogorov-Smirnov tests for the comparisons of the distributions of non-synonymous rates (dN), synonymous rates (dS), omega (= dN/dS), and percent identity conservation between the four exon-groups: constitutive with conserved (C CES) and non-conserved (C non-CES) exonic structure, and alternative with conserved (A CES) and non-conserved (A non-CES) exonic structure.

**Figure 2:**
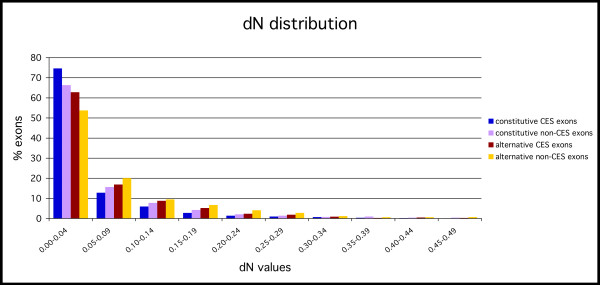
Distribution of non-synonymous rates (dN) for each of the four subsets of orthologous exons: constitutive and alternative exons with (CES) or without (non-CES) conservation of the exonic structure.

**Figure 3:**
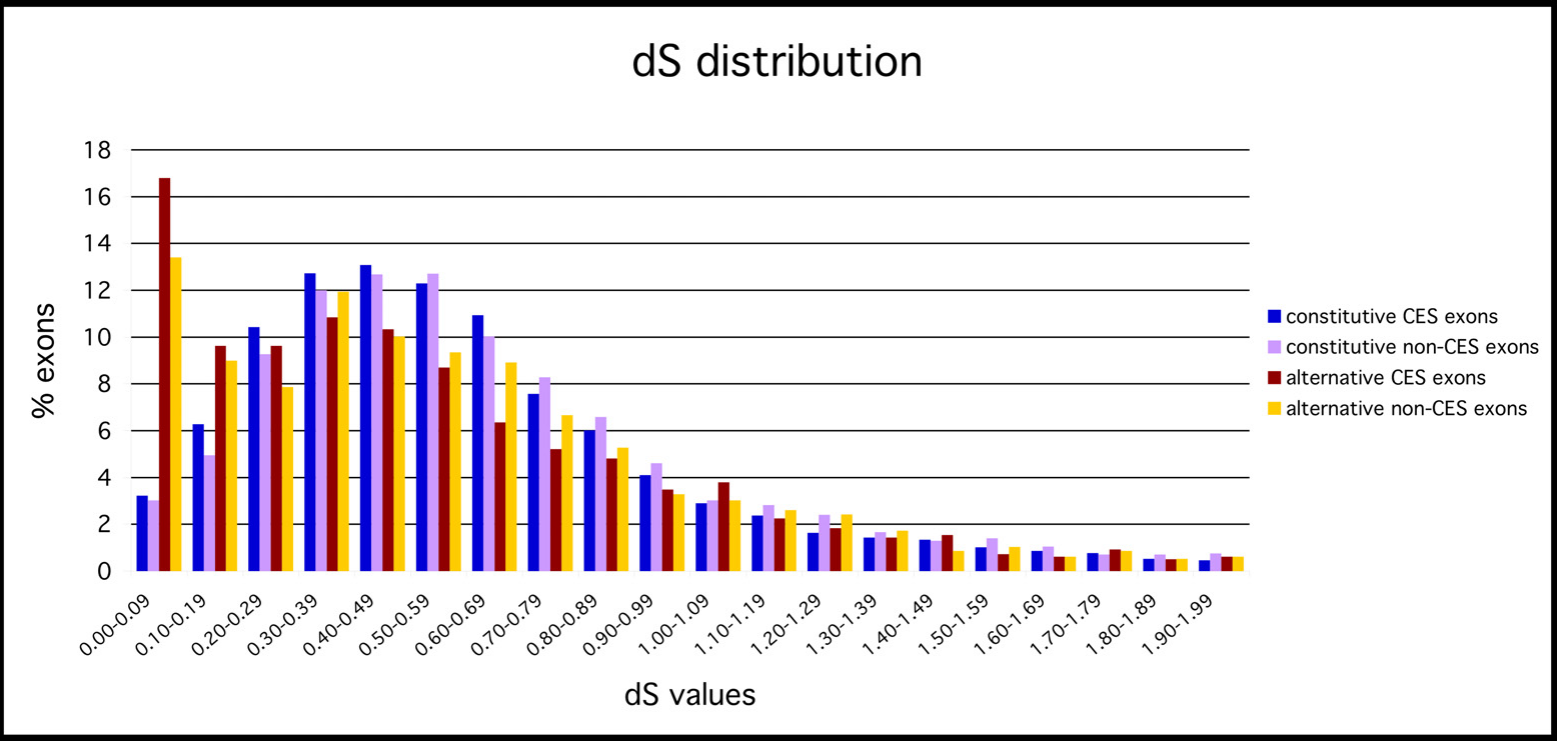
Distribution of the synonymous rates (dS) for each of the four subsets of orthologous exons: constitutive and alternative exons with (CES) or without (non-CES) conservation of the exonic structure.

**Figure 4:**
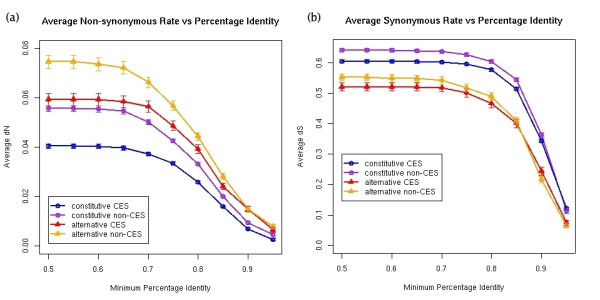
Average **(a) **non-synonymous (dN) and **(b) **synonymous (dS) substitution rates (y-axis) for different minimum percentage identity conservation values (x-axis). For each percentage identity value, we calculate the average dN, dS and corresponding standard error bars, for all the exons of each subgroup having greater or equal identity conservation.

We computed the sequence evolutionary rates for the four exon-groups and found statistically significant differences between them (Figures 2 and 3) (see Table 1 for p-values). We found that within each set of alternative or constitutive exons, non-CES exons have in general higher non-synonymous substitution rate than CES exons. Moreover, for all the exon subgroups the dN decays with the percentage identity conservation and alternative CES exons have a distribution very close to constitutive non-CES exons (Figure 4a). On the other hand, alternative non-CES exons have the highest non-synonymous rate and constitutive CES exons have the lowest non-synonymous rate. For the synonymous substitution rate we found that alternative (CES and non-CES) exons have a significantly lower synonymous substitution rate than constitutive exons (Figure 4b), and non-CES exons have a significantly higher synonymous substitution rate than CES exons. Additionally, alternative CES exons have the lowest average synonymous rate and constitutive non-CES exons have the highest one. We also found a different distribution of omega between alternative and constitutive exons after the separation into CES and non-CES exons [see Additional file 1].

We tested further the observed correlation between the evolutionary rates and the conservation of the exonic structures by looking at the differences within genes. We grouped our exons according to the gene they belong to, and selected those groups for which all the exons were of the same type, CES or non-CES. We obtained 497 groups with CES exons, and 407 with non-CES exons, where each group represents a gene. For each group we concatenated the alternative and constitutive exons into regions and compared the dN, dS and omega values for each region within each group. For the groups with CES exons we found significantly different dS and dN distributions (p-values are shown in Table 2), whereas for groups with non-CES exons we found significant differences in the dS distributions only. Interestingly, there are no significant differences in dN values between alternative and constitutive regions in groups with non-CES exons only (see Table 2). This can be explained by the observed higher dN values in non-CES exons (Figure 2) and indicates that most of the differences in dN between alternative and constitutive exons can be explained by the conservation of the exonic structure. Additionally, we found significant differences for omega between the alternative and constitutive regions of the same group for both types (CES and non-CES).

**Table 2:** P-values for the region-based comparisons of percent identity, dN, dS and omega.

	Identity	dN	dS	Omega
groups with CES exons only	2.636e-10	0.002886	34.101e-13	3.278e-14
groups with non-CES exons only	5.446e-8	0.09	6.203e-12	1.087e-10

This table shows p-values for the paired Wilcoxon signed-rank test between alternative and constitutive regions within each group, for the groups with only CES exons and the groups with only non-CES exons. Exons are grouped according to the gene they belong to.

**Figure 5:**
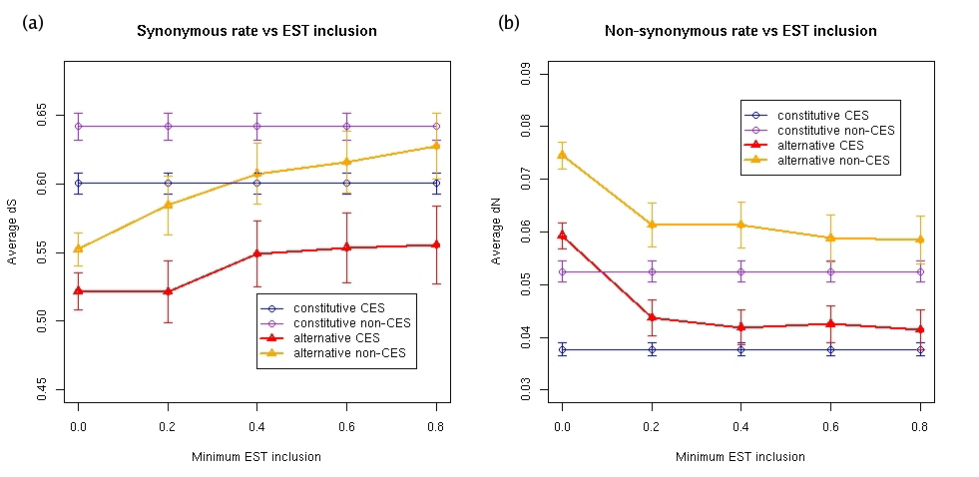
Variation of the **(a) **synonymous (dS) and **(b) **non-synonymous (dN) substitution rates for alternative CES and non-CES exons (y-axis) with the EST inclusion level (x-axis). Each average and standard error is calculated for a minimum EST-inclusion expressed as the fraction of ESTs that include the alternative exon. For comparison, we have superimposed the average synonymous rate for all constitutive CES and non-CES exons as a straight line with the corresponding error bars.

The EST-inclusion level of an alternative exon has been shown to influence the degree of cross-species conservation of the exon [4] and its substitution rate [6]. In particular, the greater the inclusion level of alternative exons, the closer the pattern of nucleotide substitution rate to that of constitutive exons. We observe a similar pattern of variation for our data set. In Figures 5a and 5b we have plotted the variation of the dS and dN with the inclusion levels for the alternative exons split into CES and non-CES exons. In this figure we have superimposed a line representing the average values of dN and dS of constitutive CES and non-CES exons for comparison. At high inclusion values, the dS values of alternative non-CES exons become indistinguishable from the dS values for constitutive exons; a KS test for the difference yielded a high p-value (0.3522). However, alternative CES exons maintain lower dS values at high EST-inclusion levels (Figure 5a). Indeed, comparing alternative CES exons of high EST-inclusion (≥50%) with the union set of all constitutive exons plus alternative non-CES of high EST-inclusion (≥50%), we found significantly different dS distributions (KS test p-value = 1.923e-05). Thus at high inclusion levels, the alternative CES exons can be separated from the rest of the exons by their average dS value.

On the other hand, each subgroup of alternative exons (CES and non-CES) approximates the average dN of the corresponding type in constitutive exons at high EST-inclusion levels (Figure 5b). However, CES and non-CES exons can still be separated. Indeed, comparing the union set of all CES exons (we take here alternative CES exons with inclusion ≥50% plus all constitutive CES exons) with the union set of all non-CES exons (we take here alternative non-CES exons with inclusion ≥50% plus all constitutive non-CES exons) we found a different dN distribution (KS test p-value = 1.782e-10). Thus at high inclusion levels, the average dN can not separate alternative and constitutive exons, but it can separate the CES from the non-CES exons.

We tested for possible biases in our classification and whether these could influence our results. We found that genes that contain non-CES exons are more frequently long and with many exons, compared to genes that contain CES exons. Using equal-sized random samples of CES and non-CES exons corresponding to the same distribution of gene lengths and exons per gene, we found the same results as reported above. Thus the gene length and the number of exons per gene do not influence our results. We also verified that the difference in the number of transcripts and exons between orthologous genes does not influence our findings. Details of this analysis are given as supplementary information [see Additional File 1].

### Evolution of exonic splicing enhancers

In order to study the evolution of the regulatory signals and its relation to the evolution of exons and exonic structures, we analyzed two sets of predicted regulatory motifs. We used 238 human [25] and 380 mouse [26] predicted exonic splicing enhancer (ESE) hexamers. These two sets were predicted independently, using the method RESCUE-ESE and without input from cross-species comparison [25,26]. We located these ESE hexamers in our set of conserved alternative and constitutive exons. We located the human predicted ESEs in the human exons and the mouse predicted ESEs in the mouse exons. Not all the ESEs in exons are conserved: 51% of the 187,084 ESEs located in the human exons were exactly conserved in sequence. On average 44% of hexamers in human coding exons are exactly conserved in mouse (data not shown). Moreover, 66% of the found ESEs were orthologous to a mouse predicted ESE, different or identical. The search of ESEs yielded a set of 20,825 *regulatory regions *in human exons. A regulatory region is defined as a range in an exon that is covered by one or more ESEs (see Methods for details).

**Figure 6:**
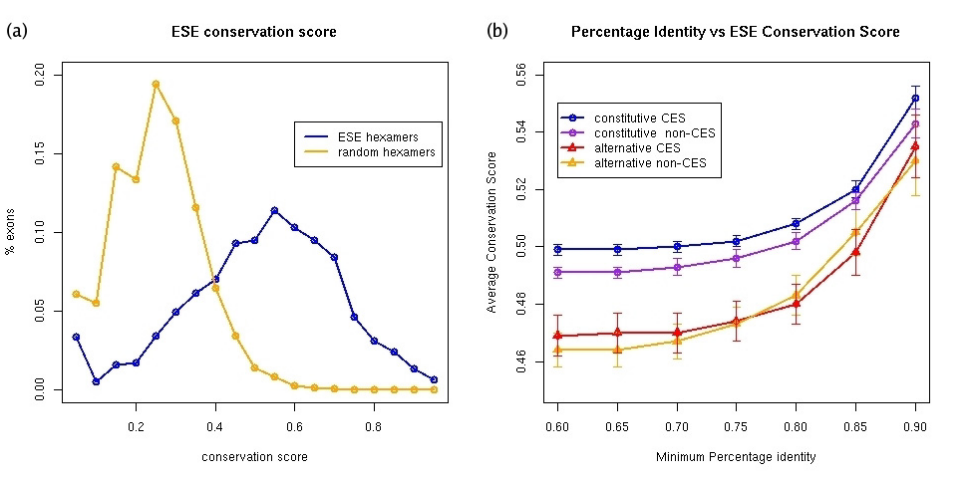
**(a) **ESE conservation score for all exons (blue) compared to the conservation score of random hexamers (orange) for the same exon set. **(b) **The ESE conservation score is plotted for each exon subgroup (alternative CES and non-CES, and constitutive CES and non-CES). The average and standard error are calculated for different minimum percent identity conservation values of the exon sequences.

Putative ESEs are found all along exon sequences [27-29]. However, we expect that relevant ESEs will have in general some positional conservation between orthologous exons with similar regulation [21]. Accordingly, we defined a *motif conservation score *as the fraction of the total region covered by motifs that is part of a motif in both human and mouse. More specifically, this is computed as the number of nucleotides (not necessarily the same) that are in a motif in human and mouse over the total length of the alignment covered by a motif in human or mouse, or both (see Methods for a detailed explanation). This score measures the equivalence between the regulatory regions in human and mouse, regardless of the sequence conservation. A conservation score of 1 means that all regions associated with splicing regulators in human are also splicing regulators in mouse. On the other hand, a conservation score of 0 means that no nucleotide associated with a regulator in human is part of a regulator in mouse. We expect orthologous exons with similar regulation to have some overlap between the regulatory regions in either exon. This overlap is not necessarily 100%, as regulators can maintain their functionality even after shifting their position. However, if this shift is too large, the functionality may be lost [21]. Thus we expect real regulators to have a conservation score much above zero. Similarly, we expect exons for which ESEs are relevant for splicing to have on average a higher conservation score. We measured the motif conservation score for the ESEs located in our exon set (Figure 6a) and found that it is higher for constitutive exons than for alternative ones (KS test p-value = 3.683e-09), as well as higher for CES exons than for non-CES exons (Figure 6b). Furthermore, the ESE conservation score decreases with increasing dS and dN, and is higher for constitutive exons for all values of dN and dS [see Additional File 1]. For comparison, we generated a set of random hexamers selected from the CDS of single-exon genes that are conserved between human and mouse. The hexamers selected were such that they have a conservation frequency in mouse similar to that found for the set of ESEs in our exon set (see Methods for details). Figure 6a shows that predicted ESEs have higher motif conservation score than the random hexamers.

**Figure 7:**
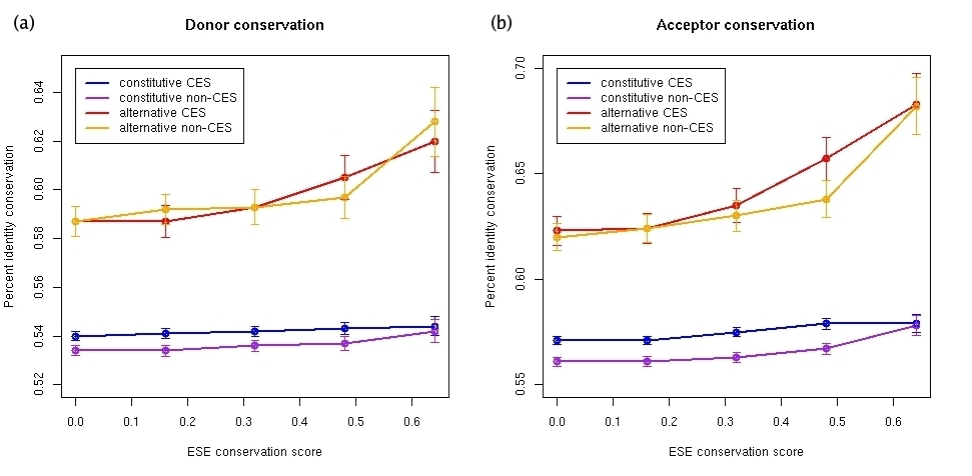
Average percent identity conservation in 100 bp of the donor **(a) **and acceptor **(b) **sites for constitutive and alternative exons plotted against the ESE conservation score. For each exon subgroup, the average conservation and standard error are calculated for different minimum ESE conservation score values.

Finally, we analyzed the conservation of introns flanking constitutive and alternative exons, performing the alignment of 100 bp at the donor and acceptor sites (Figures 7a and 7b). We observed that for constitutive (CES and non-CES) exons, the donor and acceptor conservation is approximately constant for all values of the ESE conservation score, whereas for alternative (CES and non-CES) exons the donor and acceptor identity conservation increases considerably with increasing values of the ESE conservation score. Comparing the distributions of identity conservation in introns from two groups of alternative exons, one with low ESE conservation score (< 0.20) and the other with high conservation score (> 0.60), we found differences for the donor (KS test p-value = 0.05) and acceptor (KS test p-value = 0.0017) sites. Thus at high ESE conservation score, alternative CES-exons have a significant increase in the conservation of flanking intron sequence.

## Conclusion

Using a large set of alternative and constitutive orthologous exons in human and mouse, we have revisited the analysis of the evolutionary rates in alternatively spliced regions. The results we found are consistent with the recent literature: a purifying selective pressure against silent mutations and a diversifying selective pressure for amino acid change in the alternatively spliced protein-coding regions of genes. After separating the exons according to the conservation of the exonic structure, we found that most of the differences in evolutionary rates can be explained by the pattern of conservation of the exonic structure. In particular, we have found that constitutive exons with non-conserved exonic structure (non-CES) have greater non-synonymous (dN) and synonymous (dS) sequence divergence than those with conserved exonic structure (CES). Likewise, alternative non-CES exons have greater sequence divergence than alternative CES exons. These results generalize recent findings [24] and indicate that a high divergence rate is in general linked to a variation of the transcript exonic structure. Additionally, we have found that alternative CES exons have a sequence divergence similar to that of constitutive non-CES exons, and that alternative non-CES exons have the highest non-synonymous substitution rates, whereas constitutive CES exons have the lowest. Our analyses also show that the conservation of the exonic structure is related to a constraint in synonymous divergence rate. In particular, constitutive CES exons have significantly lower synonymous rate than constitutive non-CES exons; and alternative CES exons are the most constrained in synonymous divergence, whereas constitutive non-CES exons are the least constrained. We conclude that a low dS is mainly characteristic of alternative CES-exons, and that most of the observed differences in dN between alternative and constitutive exons can be explained by the conservation of the exonic structure.

We have also observed that substitution rates in alternative (CES and non-CES) exons approximate those of constitutive exons for increasing EST-inclusion levels. The non-synonymous rate of each alternative exon type (CES or non-CES) approximates the corresponding constitutive type, such that, at high inclusion levels, alternative and constitutive exons of the same type have indistinguishable dN distributions. However, at high inclusion levels, CES and non-CES exons can still be separated by their dN. On the other hand, the synonymous rate of alternative non-CES exons increases such that at high EST-inclusion levels they cannot be separated from constitutive ones, whereas alternative CES exons keep a synonymous rate low enough to be separable from constitutive exons at high EST-inclusion levels. These findings give further indication that the differences in non-synonymous rates can be mostly explained by the differences in the pattern of conservation of the exonic structure. Furthermore, we can also conclude that a low synonymous substitution rate characterizes alternative CES exons at all inclusion levels.

The relations we have found between the evolution of sequence and exonic structure provide an explanation for the apparent contradictions between reports of a high sequence conservation of exon-skipping events [9,18,24,30] and reports about a high non-synonymous substitution rate in alternative exons [5-7]. These discrepancies can be explained by the differences in the conservation of the exonic structures. We have found that alternative exons are overrepresented in the range of high (> 95% identity) and low (< 80% identity) sequence conservation. Additionally, alternative CES exons have more sequence conservation than alternative non-CES exons; they have lower non-synonymous and synonymous substitution rates. The high sequence conservation low substitution rates in alternative exons reported previously in the literature are therefore explained if the analyzed alternative exons are part of conserved exonic structures.

Finally, we have studied the evolutionary properties of the exonic signals regulating the splicing. We localized two independent sets of human and mouse predicted exon splicing enhancers (ESEs) in our set of alternative and constitutive exons, and defined a measure of the conservation of the arrangement of regulatory signals, the *motif conservation score*. We have found that the motif conservation score for ESEs can separate constitutive exons from alternative ones: constitutive exons have on average higher motif conservation score. This indicates that the conservation of ESEs is important for maintaining the "constitutiveness" of an exon. For constitutive exons, CES exons have on average higher ESE motif conservation score, which indicates a relation between the conservation in the arrangement of splicing regulators and the conservation of the exonic structure. We also observed that alternative exons with high ESE motif conservation scores have higher sequence conservation in the flanking intron sequence. Strikingly, constitutive exons maintain the same average conservation at the flanking introns, independently of the level of conservation of the ESE-arrangement. This indicates that for constitutive exons, the regulation takes place mainly at the exon sequence. On the other hand, alternative exons with high sequence conservation have a pattern of ESE-arrangement conservation very similar to constitutive exons and have a conservation at the flanking introns greater than average, possibly due to a higher density of conserved regulatory motifs [32]. This indicates that highly conserved alternative exons must be strongly regulated by intron signals, since their pattern of ESE conservation is very similar to that of constitutive exons. This may also explain the high sequence conservation previously observed at the introns flanking conserved skipped exon events [30,31]. Finally, our analyses show that the low conservation observed in cross species alignments of transcript exonic structures between human and mouse [33] is intimately related to the evolution of the exon sequences and the regulators of splicing. We expect our results will be of help for the prediction of novel transcript structures using cross-species comparisons [33,34].

## Methods

### Exon data sets

We extracted the Ensembl annotations for human (NCBI35, build 124) and mouse (NCBIM33, build 124), and the EST alignments from the UCSC browser (Dec 2004). EST alignments were processed to obtain an exonic structure for each EST and only spliced alignments were used. Comparing the Ensembl annotations to the ESTs we extracted a set of constitutive and cassette exons in human and mouse. The EST data was only used to deduce the constitutive or alternative nature of the exon, and all exons used were from annotated Ensembl CDSs, which are based on protein and mRNA data [35]. Alternative 5' or 3' exons were not included in the set. Using the set of unique-best-reciprocal-hit orthologous genes from Ensembl [36], we compared the exon sequences within each orthologous gene pair with exonerate [37] to obtain a set of orthologous exon pairs. The exon pairs were separated into four sets, according to whether both, either or none of the exons were skipped. This resulted in 10,005 orthologous exon pairs for which both exons were constitutive and 2,724 exon-pairs for which both exons were alternative. We performed the evolutionary analysis on a subset of these (see below).

### Study of synonymous and non-synonymous substitution rates

We performed a global alignment using ClustalW [38] of the coding sequence of every orthologous exon pair. Frameshifts between the sequences were allowed and the stop codons were removed from all the sequences. To calculate synonymous and non-synonymous divergence we employed the 'codeml' application (runmode = -2, CodonFreq = 2) in the PAML package [39]. This method performs a Maximum Likelihood analysis to calculate dS, dN and omega (dS/dN) in coding sequences. The human-mouse orthologous exon pairs and their values of dN, dS and omega are available as supplementary material [see Additional file 2].

In order to compare the dN, dS and omega values between alternative and constitutive exons we performed a Kolmogorov-Smirnov test. For the analysis of regions, we performed Wilcoxon signed-ranks test to check if the distributions in the per-gene comparisons were equal or not. The tests were performed with the R statistical package [40].

We calculated dN, dS and omega for all the genes in two different ways. First, for each gene we concatenated together the sequence of all the alternative exons on one hand and of all constitutive exons on the other hand, and calculated the difference in dN, dS and omega between constitutive and alternative sequences for each gene. In the second approach, we calculated dN, dS and omega for every exon. Moreover, we considered only those exons that had dS ≤ 2 and dN ≤ 0.5. These constraints were enforced to ensure that the exons were real orthologs. For the per-gene comparisons, only those genes for which all the exons had dS ≤ 2 and dN ≤ 0.5 were analyzed. For the analysis of dN and dS for the exons separately, 2133 alternative exons and 8788 constitutive exons were included. For the omega analysis 90 alternative exons and 41 constitutive exons were removed because they had omega undefined. For the analysis of dN and dS using the concatenated sequences 1072 genes were considered, and for the analysis of omega 35 genes were removed because they had omega not defined.

### Exon subgroups

We have classified the alternative and constitutive exons into four groups according to whether they are part of a transcript with conserved exonic structure (CES) or not (non-CES): constitutive CES and non-CES, and alternative CES and non-CES. A non-CES exon is such that there is a pattern of splicing of the pre-mRNA, which includes this exon, and which is never the same in the orthologous pre-mRNA when the orthologous exon is included. An exon is considered to be of CES type if there is a transcript to which belongs such that there is a transcript in the orthologous gene that contains the exon and has very similar exonic structure. As the exonic structure is conserved, the splicing regulation must be conserved, and in particular, the orthologous exons must have similar splicing regulation. The criterion chosen to determine the conservation of exonic structures is as follows: both transcripts must have at least three exons and the number of exons can differ at most by one. Performing a global alignment where the aligned symbols are the exons, there cannot be internal gaps (missing exons) and aligned exons must have the same phase. Using this criterion we obtained 4688 constitutive CES exons, 4100 constitutive non-CES exons, 977 alternative CES exons and 1156 alternative non-CES exons.

### Motif conservation score

**Figure 8:**
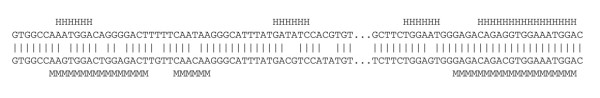
Alternative exon (CES type) of the chloride channel 6 isoform CIC-6a (CLCN6, ENSG00000011021) gene aligned to the orthologous exon in mouse. We have omitted 13 bp of alignment that does not contain any ESEs. The H's and M's indicate the human and mouse *regulatory regions*, respectively.

In order to illustrate the calculation of the conservation score, we consider the example shown in Figure 8. A regulatory region is defined as a piece of exon covered by one or more ESEs. We call *N *the total length of the alignment covered by human and mouse ESEs, i.e., the number of aligned nucleotides labelled as H and M in Figure 8. Similarly, we call *M *the total length of the alignment covered by a motif in human or mouse, or both, i.e., the number of aligned nucleotides labelled as H or M, or both, in Figure 8. The *motif conservation score S *is therefore defined as *S *= *N*/*M*. For the example in Figure 8, *N *= 22 and *M *= 54, hence the motif conservation score for this exon is 0.4.

### Random hexamers

Our aim was to compare the arrangement of the ESE motifs with the arrangements of hexamers that have similar frequency of conservation (see Figure S6 of Additional File 1) and participate in coding sequences. We did not impose any nucleotide content bias, as ESEs are purine-rich [24] and this property alone may be enough to characterize a region as having potential splicing enhancing functionality. We therefore extracted hexamers and their conservation frequencies from the CDS of single-exon genes conserved between human and mouse. Known genes annotated with a single-exon and a complete CDS were extracted from Ensembl for human and mouse. We aligned the CDSs using ClustalW, and only the 536 cases with a correct alignment of the start and stop codons were kept. We extracted the 3391 hexamers occurring in these human exons and computed the fraction of exact conservation for these hexamers in the alignments. Hexamers containing N's or any other ambiguity code were rejected. From the obtained hexamers, we selected 10 times two independent random sets of 238 and 380 hexamers, respectively, with a conservation frequency similar to that of ESEs [see Additional File 1]. The motif conservation score was calculated for the exons in our data set, using the ESEs and the 10 sets of random hexamers. The distribution of these scores can be seen in Figure 6a.

### Intron alignments

We extracted 100 bp from the intronic sequences flanking our sets of exons. Orthologous sequences were aligned with ClustalW, and the percentage identity conservation was calculated from the alignment. Alignments with a misaligned GT or AG di-nucleotide at the donor or acceptor sites, respectively, were rejected.

## Authors' contributions

MP carried out the analysis of the evolutionary measures for the different exon sets and contributed to the writing of the manuscript. EE did the analysis of the regulatory sequences, coordinated the research and led the writing of the manuscript. Both authors read and approved the final manuscript.

## Supplementary Material

Additional File 1 File with supplementary tests and information. Click here for file

Additional File 2 File with the data used to perform the analysis. Click here for file
