# Supplementary material for: Differentiated evolutionary rates in alternative exons and the implications for splicing regulation
Source: BMC Evol Biol. 2006 Jun 22;6:50. doi: 10.1186/1471-2148-6-50 (PMC1543662; doi:10.1186/1471-2148-6-50)
Supplement: Additional File 1 — File with supplementary tests and information. [file 1471-2148-6-50-S1.pdf]

Supplementary material - Differentiated evolutionary rates in alternative exons and the implications for splicing regulation

Mireya Plass[1], Eduardo Eyras[1,2,3]

[1]Research Unit of Biomedical Informatics, IMIM - Pompeu Fabra University, E08003, Barcelona, Spain
[2]Catalan Institution for Research and Advanced Studies (ICREA), E08010, Barcelona, Spain

[3]Corresponding author: eduardo.eyras@upf.edu

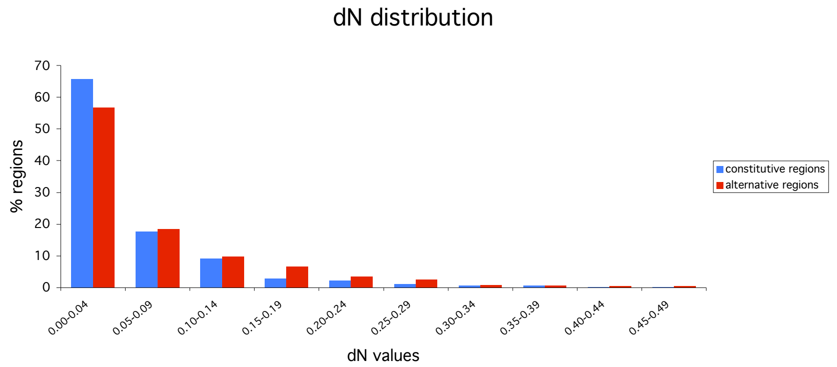

Figure S1. Distribution of the non-synonymous substitution rate (dN) for constitutive and alternative regions.

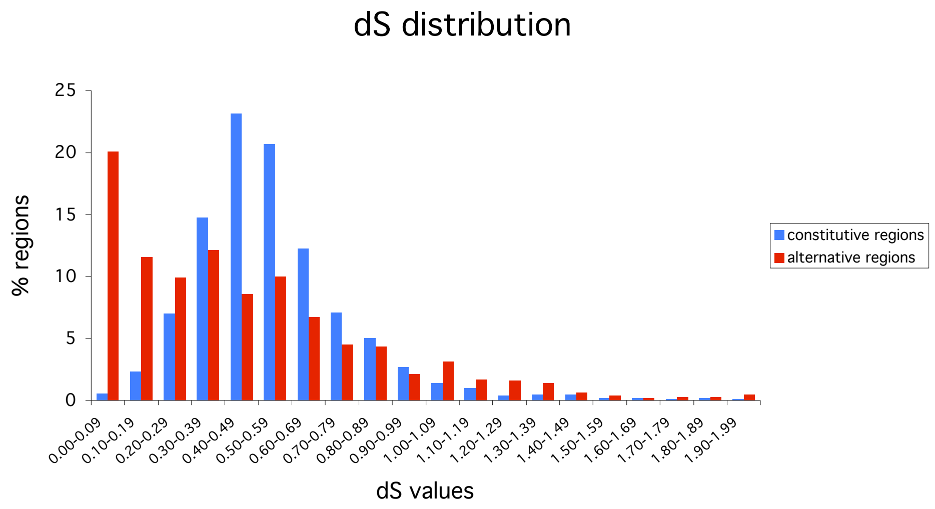

Figure S2. Distributions of the synonymous substitution rate (dS) for constitutive and alternative regions.

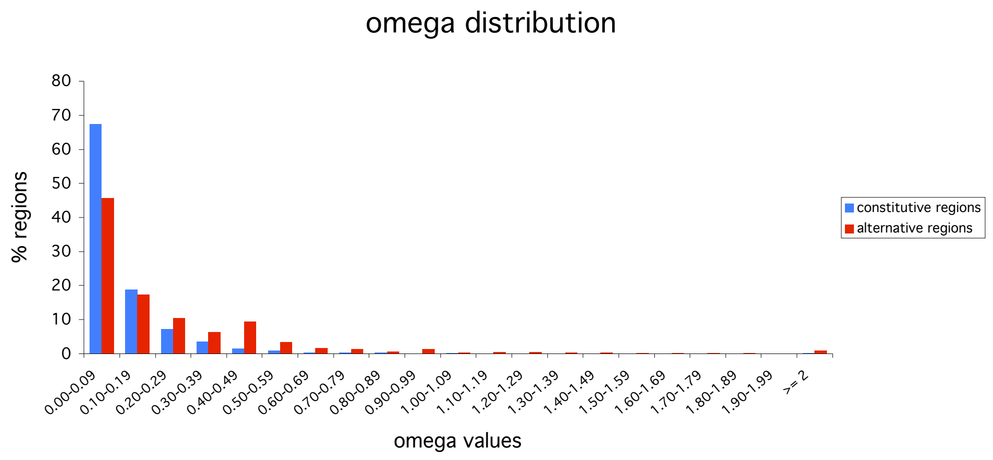

Figure S3. Distribution of the values of Omega (=dN/dS) for the alternative and constitutive regions. Alternative (red) and constitutive (blue) exons have significantly different distributions (p-value < 2.2e-16).

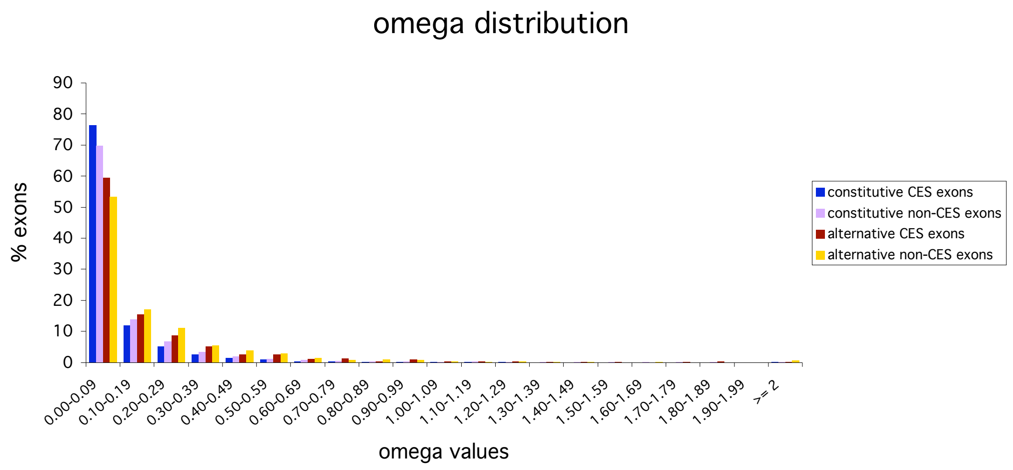

Figure S4. Distribution of omega (=dN/dS) for each of the four subsets of orthologous exons: constitutive and alternative exons with (CES) or without (non-CES) conservation of the exonic structure.

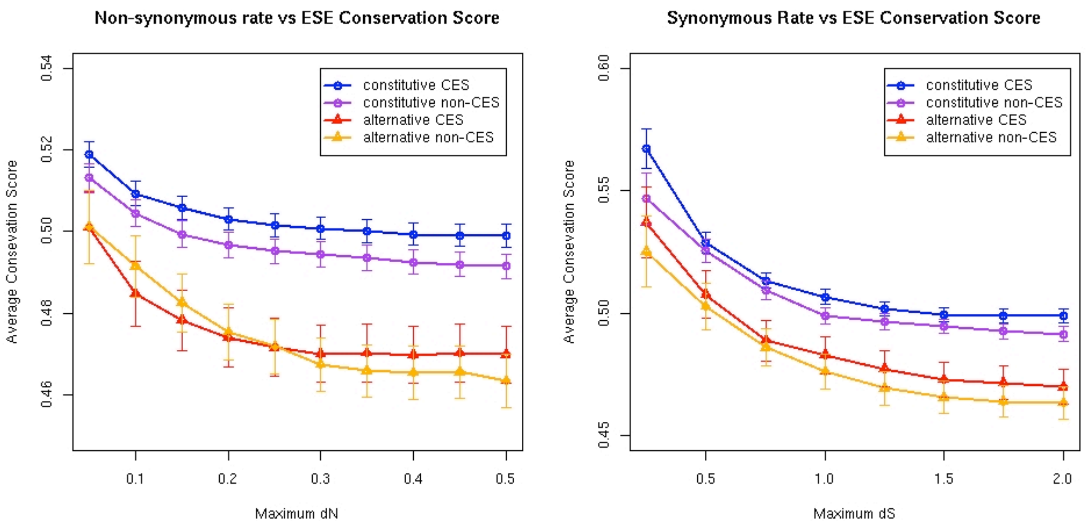

Figure S5. Correlation of the ESE conservation score with the (left) non-synonymous (dN) and (right) synonymous (dS) divergence for each of the four exon-groups.

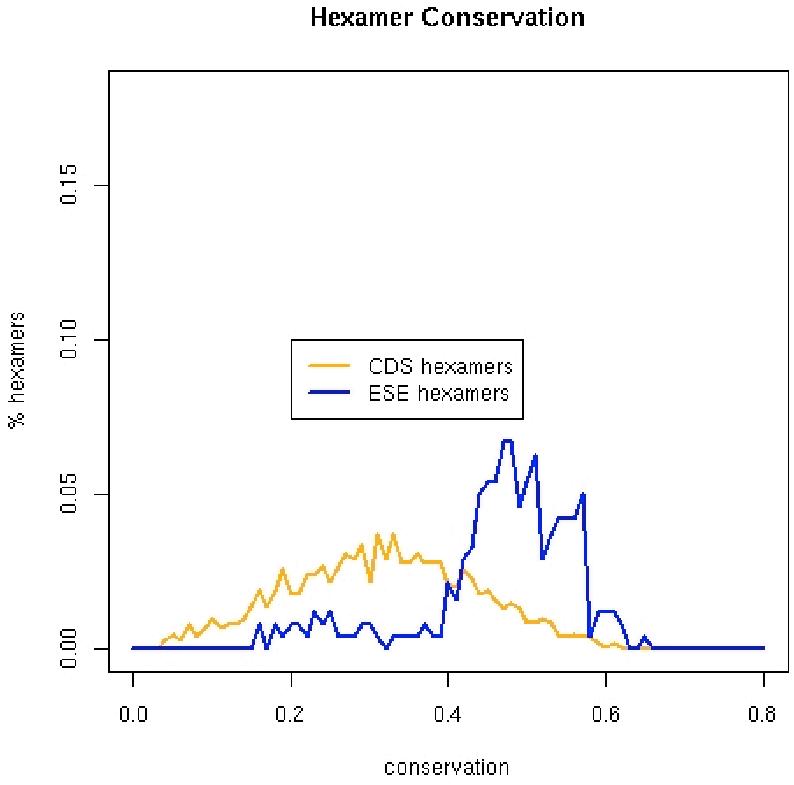

Figure S6. Distribution of the conserved hexamers for two exon data sets: hexamers in the CDS of single exon genes (orange) and ESE hexamers from our set

of constitutive and alternative exons. On the x-axis, the conservation is given as the fraction of the occurrences of the hexamers in human that is exactly conserved in mouse. The y-axis represents the proportion of hexamers with a given conservation.

Density of Exonic Enhacers in Alternative and Constitutive exons

We compared the density of ESEs in constitutive and alternative exons. For each gene we calculate the difference in the proportion of bases covered by ESEs in constitutive and alternative exons:

$$\left.\frac{bp_{ESEs}}{bp_{exons}}\right|_{const} - \left.\frac{bp_{ESEs}}{bp_{exons}}\right|_{alt}$$

We found a higher density of ESEs in constitutive exons. The mean of the differences is 0.016. A check of the difference using paired t-test gives a p-value = 6.273e-05, and a 95% confidence interval [0.008496871, 0.024703948], which is not overlapping 0. From this we can conclude that constitutive exons have a slightly higher density of ESEs.

Further, we plotted the density of ESEs in alternative and constitutive exons separated by CES and non-CES exons (Figure S8). This average density was plotted for each exon subset, at different minimum percentage identity values. Slicing the data in this way, we can view the differences between the sets, and how these differences change with the conservation. We observe that constitutive exons have in general higher density of ESEs than alternative exons.

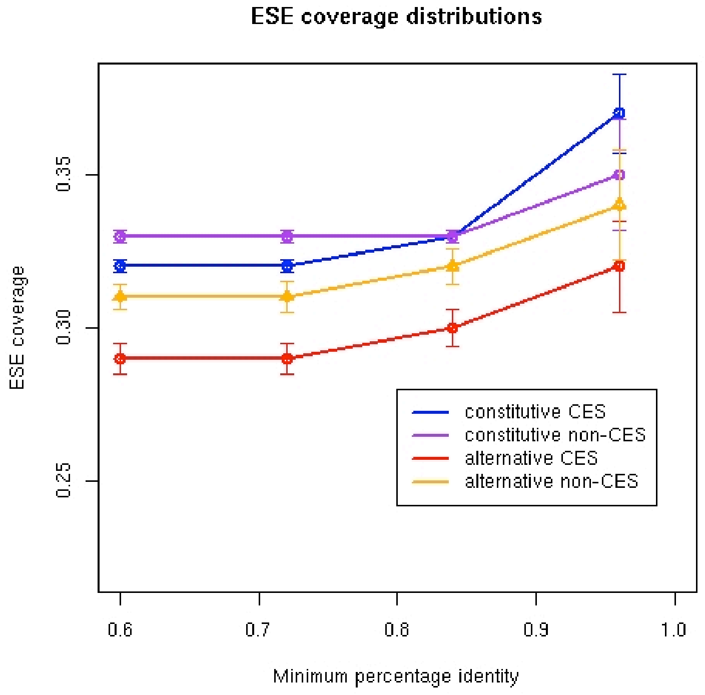

Figure S7. Correlation of the ESE density versus the minimum percentage identity conservation of exon sequences. The ESE density is measured as the fraction of the exon length in human that is covered by ESEs.

Testing the influence of biases in the results

We wanted to test whether there are biases in our dataset and whether these could influence the results that we present in our paper. For this work, we had classified our exon set according to whether they appear in a transcript with an exonic structure that is conserved (CES) or not conserved (non-CES) between human and mouse. In order to test the influence of possible biases we have considered the genes to which these exons belong, and separated them into two sets: those containing conserved exons (that we call CES-exon-containing genes) and those containing non-CES exons (that we call nonCES-exon-containing genes), and considered the distributions of the number of exons per gene, gene length, and difference in the number of transcripts between gene orthologs. We found that there are some characteristics more typical of genes containing non-CES exons, but none of these properties influence the results we present in our manuscript. A detailed explanation of this analysis is given below.

Dependencies with the number of exons per gene

We compared the distributions of the number of exons per gene for CES-exon-containing genes and nonCES-exon-containing genes. We observe that nonCES-exon-containing genes are more frequent in the range of 22 or more exons per gene, whereas CES-exon-containing genes are more frequent in the rage of less than 22 exons per gene (see Figure 1).

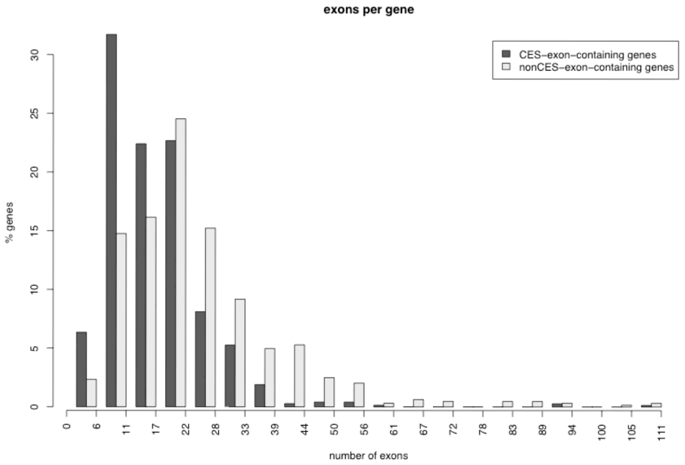

Figure 1. ***Distributions of the number of exons per gene for CES-exon-containing genes and nonCES-exon-containing genes.***

To test whether these biases have any influence in the differences of dN and dS that we observe for the four different exon subtypes (constitutive CES, constitutive non-CES, alternative CES and alternative non-CES), we did a equal-sized random sampling of exons from this distribution. More specifically, from the distribution of the number of exons per gene we considered the following 5 bins:

number of exons-per-gene	constitutive CES exons	constitutive non-CES exons	alternative CES exons	alternative non-CES exons
3-11	942	169	291	112
12-17	915	353	215	110
18-23	1293	715	233	222
24-29	580	690	111	142
30-40	767	836	108	241

In the table we include the number of exons of each exon-subset present in each of these bins. These bins account for the 82.8% of the total number of exons considered in the paper.

From each bin, and from each exon-subtype we sampled 20 exons at random, hence 100 exons for each exon subtype, and calculated the average dN and average dS values for each subtype. This random sampling and average calculation was repeated 10000 times. Figure 2 shows the distribution of the average dN values from this 10000 samplings for each exon subtype. We observe the same behaviour reported in the manuscript: non-CES exons have higher dN than their CES counterparts. In particular, alternative non-CES exons have on average the highest dN values, whereas constitutive CES exons have on average the lowest dN values.

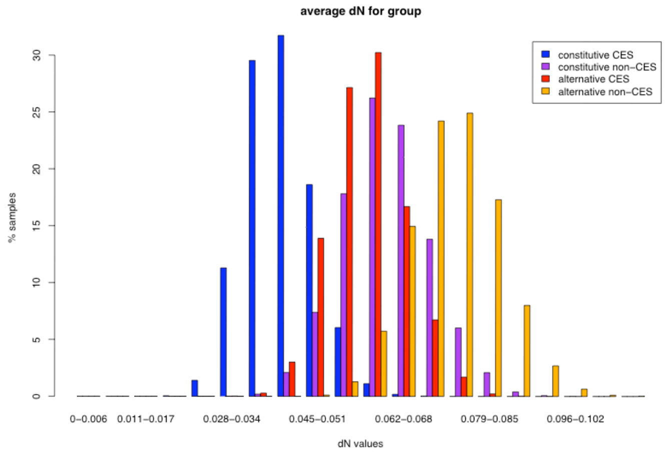

Figure 2. ***Distribution of the average dN values for the four exon sets (constitutive CES, const. non-CES, alternative CES and alt. non-CES), obtained from an equal-sized random sampling of equivalent bins of the exons-per-gen distribution.***

Figure 3 shows the distribution of the average dS values for each exon subtype, and reflect the same pattern described in the manuscript: CES exons have lower average dS values. In particular, alternative CES exons have on average the lowest dS values, whereas constitutive non-CES exons on average the highest dS values.

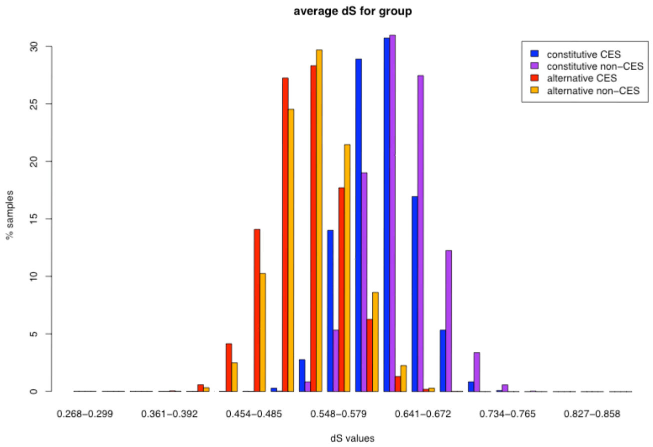

Figure 3. ***Distribution of the average dS values for the four exon sets (constitutive CES, const. non-CES, alternative CES and alt. non-CES), obtained from an equal-sized random sampling of equivalent bins of the exons-per-gen distribution.***

We can conclude that the number of exons per gene does not affect our results.

Dependencies with the gene length

The gene-length distributions follow the same trend as for the number of exons per gene. Short genes are more frequently CES-exon-containing than nonCES-containing, and long genes are more frequently nonCES-exon-containing than CES-exon-containing ones (see Figure 4).

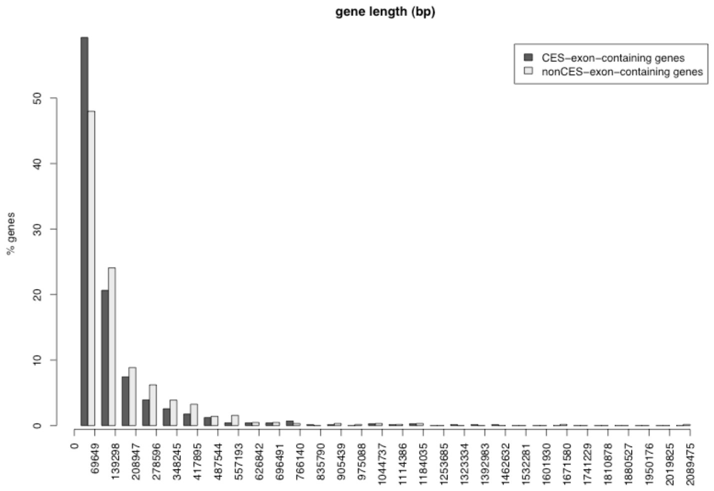

Figure 4. ***Distributions of the gene-lengths for CES-exon-containing genes and nonCES-exon-containing genes.***

We performed the same random sampling procedure of equal-sized exon subsets as before, now using the gene-length distribution. We considered the following bins:

gene lengths (bp)	constitutive CES exons	constitutive non-CES exons	alternative CES exons	alternative non-CES exons
0-72051	2286	1391	529	508
72052-144102	1100	1023	240	283
144103-216153	449	479	69	113
216154-288203	397	573	62	142
360254-648458	279	535	49	65

In the table we also give the number of exons for each subtype. These bins account for the 96.8% of the total number of exons used in the paper.

As before, from each bin and for each exon-subtype, we sampled 20 exons at random, hence 100 exons for each subtype. This was repeated 10000 times, and each time, the average dN and dS for each subtype was calculated. We obtained the same results as before: non-CES exons have on average higher dN values (see Figure 5) and CES-exons have on average lower dS values. (see Figure 6). We therefore conclude that the gene length does not affect our results.

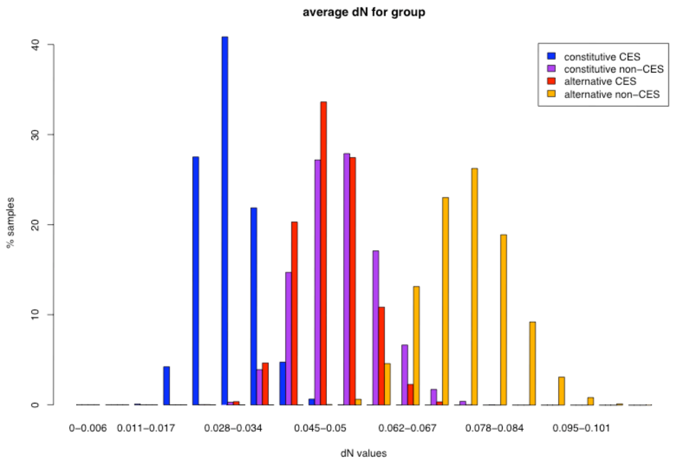

Figure 5. ***Distribution of the average dN values for the four exon-subtypes (constitutive CES, const. non-CES, alternative CES and alt. non-CES), obtained from an equal-sized random sampling of equivalent bins of the gene-length distribution.***

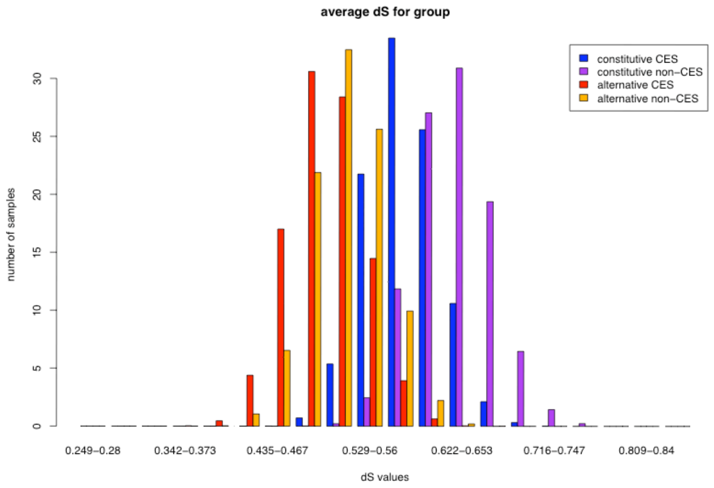

Figure 6. ***Distribution of the average dS values for the four exon-subtypes (constitutive CES, const. non-CES, alternative CES and alt. non-CES), obtained from an equal-sized random sampling of equivalent bins of the gene-length distribution.***

Dependencies with the differences in the number of transcripts per gene between orthologous pairs

We also looked at the possible dependencies with the difference in the number of transcripts in human and mouse gene orthologs. For each pair of human-mouse gene orthologs, we calculated the distribution of the differences in the number of transcripts. The distributions for CES-exon-containing and nonCES-exon-containing genes are shown in Figure 7.

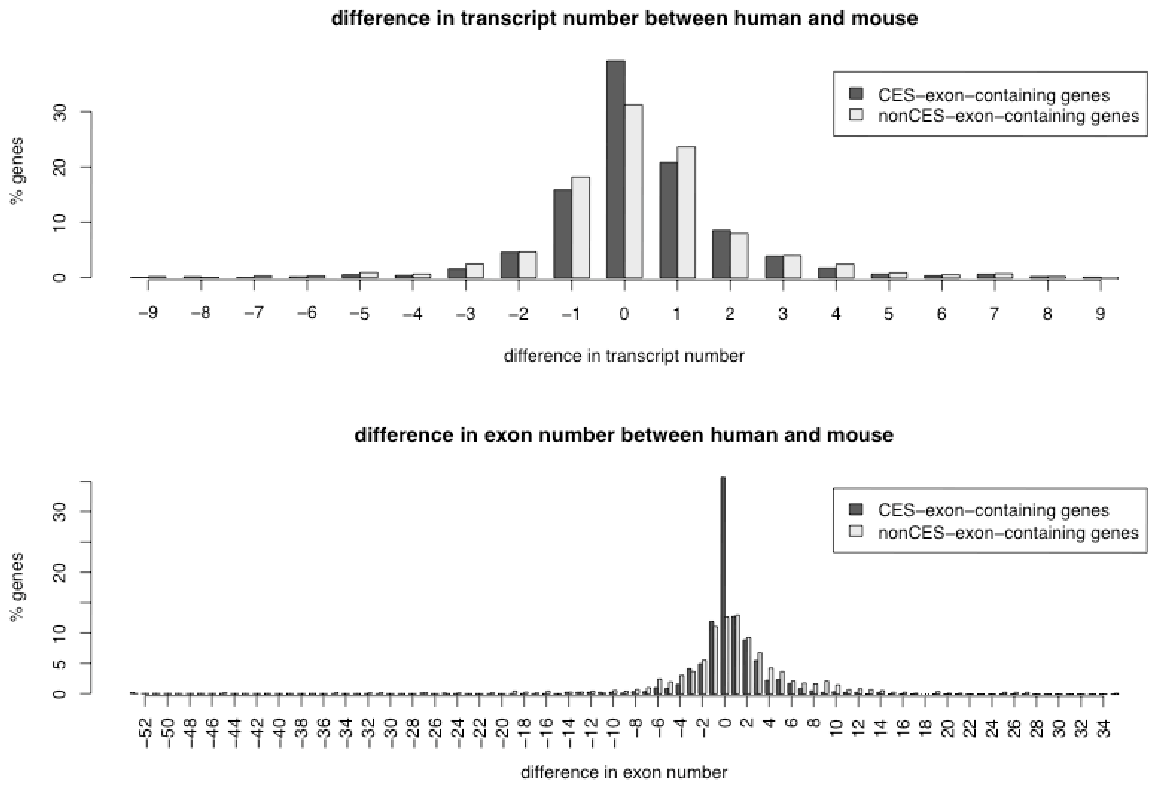

Figure 7. ***Distributions of the differences in the number of transcripts (above) and in the number of exons (below) between human-mouse orthologous gene-pairs. The x-axis is calculate subtracting the number in mouse to the number in human.***

To test whether our results are influenced by the cases in which there is a big difference in the number of transcripts between orthologous genes, we calculated the distributions of dN and dS for orthologous pairs that have the same number of transcripts (see Figure 8). For this subset, the CES-exon-containing genes have on average 1.8 transcripts (median 2), and the nonCES-exon-containing genes have on average 2.3 transcripts (median 2).

We observe (see Figure 8) that the exons distribute with the same general trend as reported in the manuscript: constitutive CES exons have lower dN values, alternative non-CES exons have higher dN values, alternative CES exons have lower dS values and constitutive non-CES have higher dS values.

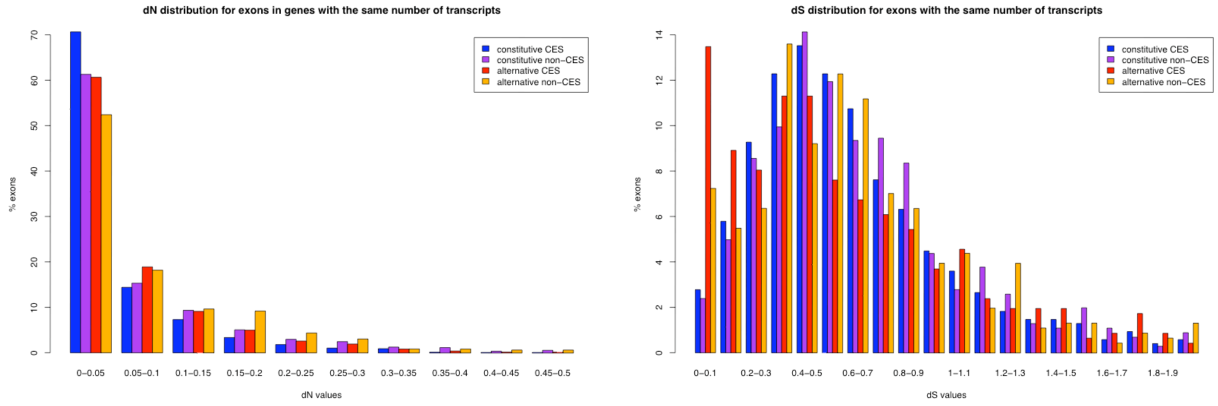

Figure 8. ***Distribution of the dN and dS values for exons (separated in four exon subtypes) in genes orthologs with the same number of transcripts per gene.***

We looked at the distribution of the differences in the number of exons per gene between human and mouse orthologs (see Figure 7). We clearly see that orthologous genes with the same number of exons contain more frequently CES exons. This, however, is an expected feature of our classification: we want to distinguish between cases where the exonic structure varies and cases where it does not. This variation is expected to correlate with gene orthologs with exons that are species specific. Thus orthologous genes with the same number of exons are more likely to share most of the exonic structures. We also note that we are considering only coding exons in our analyses.

We calculated the dN and dS distributions for the different exon-subsets in the case where the orthologous genes have the same number of exons (see Figure 9). We observe that the exons distribute with the same general trend as reported in the manuscript: constitutive CES exons have lower dN values, alternative non-CES exons have higher dN values, alternative CES exons have lower dS values and constitutive non-CES have higher dS values.

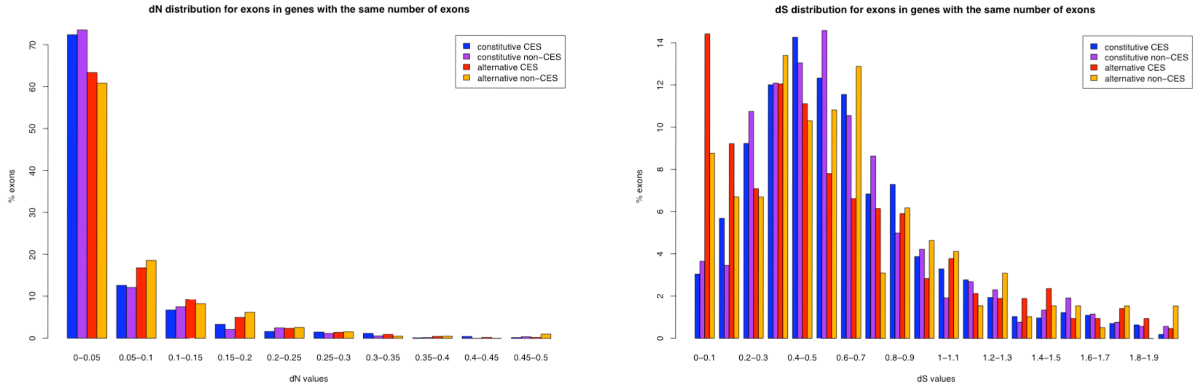

Figure 9. ***Distribution of the dN and dS values for exons (separated in four subtypes) in genes orthologs with the same number of exons per gene.***

Conclusions

We have sliced our data taking into account that a number of genes contain one or more transcripts which exonic structure is not conserved in mouse. The present tests show that these genes are quite often long and with many exons. We also show that, however, these features do no influence the findings reported in our article. We therefore can expect that long genes with many exons are more prone to vary in exonic structure with respect to their orthologs. However, independently of the type of gene in which this variation is more frequently observed, it is the variation itself what correlates with a difference in sequence conservation. Thus we can conclude that the sequence properties of alternative exons depend on contextual factors. A subset of alternative exons has higher sequence conservation than average, and a different subset has higher dN than average, and these subsets strongly correlate with exons in conserved and non-conserved exonic structures, respectively.
